# Efficacy of Intense Pulsed Light AOPT‐LTL Technique in the Treatment of Melasma: An In Vivo and Clinical Study

**DOI:** 10.1111/jocd.70443

**Published:** 2025-09-08

**Authors:** Jing Wang, Xinlan Wang, Li Gu, Zhinan Shi, Zhiyi Xu, Siqi Shen, Liqun Gu, Lin Chen, Linling Ju, Chenglong Jin, Bingrong Zhou, Hui Hua

**Affiliations:** ^1^ Department of Dermatology, Nantong Third People's Hospital Affiliated Nantong Hospital 3 of Nantong University Nantong China; ^2^ Medical School of Nantong University Nantong China; ^3^ Department of Dermatology Ren Ji Hospital, Shanghai Jiaotong University School of Medicine Shanghai China; ^4^ Nantong Institute of Liver Diseases, Nantong Third People's Hospital Affiliated Nantong Hospital 3 of Nantong University Nantong China; ^5^ Department of Dermatology Suzhou Mylike Cosmetic Hospital Suzhou China; ^6^ Department of Dermatology The First Affiliated Hospital of Nanjing Medical University Nanjing China

**Keywords:** AOPT‐LTL, IPL, mast cells, melasma

## Abstract

**Purpose:**

To evaluate the efficacy and underlying mechanism of advanced optimal pulse technology intense pulsed light (AOPT) in low‐energy triple‐pulse long‐width mode (AOPT‐LTL) for melasma treatment.

**Methods:**

An in vivo guinea pig model of melasma was established through progesterone injection and ultraviolet B radiation. Three sessions of AOPT‐LTL treatment were performed weekly. The mRNA levels of key melanogenic enzymes, inflammatory factors, pro‐angiogenic cytokines, and collagenolytic proteases were detected by qRT‐PCR. Protein levels of stem cell factor (SCF) and mast cell growth factor receptor (c‐KIT) were detected by immunofluorescence staining and Western blotting. Twenty melasma patients were treated with three sessions of AOPT‐LTL treatment monthly, evaluated by the Modified Melasma Area and Severity Index (mMASI) and Erythema Index (EI).

**Results:**

In guinea pigs with melasma, AOPT‐LTL treatment effectively mitigated pigmentation and suppressed the expression of key genes involved in melanin production. There was a reduction in mast cell infiltration, decreased expression of inflammatory factors and pro‐angiogenic factors, inhibition of angiogenesis, and alleviation of skin photoaging. Furthermore, AOPT‐LTL also diminished the expression level of the SCF/c‐KIT ligand/receptor pathway, which is closely associated with mast cell proliferation and activation. Consistently, three sessions of AOPT‐LTL treatment effectively reduced skin melanin content, erythema severity, as well as the mMASI and EI scores in melasma patients.

**Conclusions:**

AOPT‐LTL treatment significantly enhances the improvement of melasma by diminishing melanogenesis, inflammation, angiogenesis, mast cell infiltration, and collagen degeneration, potentially through the inhibition of the SCF/c‐KIT ligand/receptor pathway.

## 1 Introduction

Melasma is acquired facial pigmentation characterized by extensive and symmetrical butterfly‐like or irregular light brown to dark brown patches on the zygomatic cheek, periorbital region, forehead, upper lip, nose, and other facial regions. The incidence of melasma varies with race and gender, particularly high (40%) in dark‐skinned races. Melasma is more prevalent in women, especially those of childbearing age [1], or young and middle‐aged individuals. Due to its higher sensitivity to ultraviolet rays, light‐colored skin is more subjected to melasma [2]. Besides facial pigmentation, melasma frequently induces low self‐esteem, social barriers, negative emotion, and low quality of life [3], thus necessitating the exploration for effective prevention and treatment methods.

The pathogenesis of melasma is complicated and associated with genetic susceptibility, fluctuation of estrogen levels, and long‐term ultraviolet exposure [4, 5, 6]. Histopathologically, melasma is manifested as hyperpigmentation in the dermis, hyperfunction of melanocytes, overproduction of melanosomes, hypervascularity in the dermis, mast cell infiltration, and elastin degeneration [7, 8, 9]. Under ultraviolet irradiation, mast cells are stimulated to promote facial pigmentation via releasing histamine and cytokines in the dermis, as well as boosting the proliferation and migration of melanocytes [10]. Additionally, mast cells induce vascular proliferation by secreting pro‐angiogenic factors like vascular endothelial growth factor (VEGF), fibroblast growth factor‐2 (FGF‐2) and transforming growth factor‐β (TGF‐β) [11]. Previous studies have confirmed the regulation of mast cells on photoaging. Trypsin, released by mast cells under long‐term UV irradiation, directly causes elastin degeneration and indirectly induces the degradation of dermal collagen and extracellular matrix through activating pro‐matrix metalloproteinase [12].

Treatment options vary between the active and stable stages of melasma. Systemic applications of tranexamic acid, glycyrrhizin, and glutathione, supplemented by topical hydroquinone and its derivatives, tretinoin, azelaic acid, and tranexamic acid, are preferred in the active stage. In the stable stage, chemical peeling and photoelectric therapy can be additionally performed. However, long‐term systemic and topical treatment results in adverse events, such as coagulation anomalies, gastrointestinal dysfunction, and local irritation [13]. Using an incoherent broad‐spectrum light with a wavelength ranging from 400 nm to 1200 nm, intense pulsed light (IPL) is a phototherapy for skin pigmentation, vascular diseases, hair removal, and skin rejuvenation [14]. IPL has shown effectiveness for melasma [15, 16], although the clinical application has been plagued by the relapse of pigmentation events due to an aggressive selection of treatment parameters [17]. Photobiomodulation (PBM), relying on laser and incoherent light, can achieve the goal of regulating physiological functions. It is characterized by noninvasiveness, low energy, and non‐thermal effect. Dai et al. [18] reported the role of PBM, using the light‐emitting diode (LED) light at the wavelength of 590 nm, in reducing pigmentation and erythema of melasma patients. Our previous study has validated the excellent therapeutic efficacy of IPL combined with PBM at an innovative mode of advanced optimal pulse technology with low energy, three pulses, and long pulse width (AOPT‐LTL) in treating rosacea [19]. However, whether this new treatment mode is effective in the treatment of melasma and the specific mechanism is still unclear.

In the present study, we first examined the efficacy of AOPT‐LTL treatment in treating melasma and the underlying mechanisms in a guinea pig model. Furthermore, this efficacy was validated in a cohort of 20 melasma patients.

## 2 Methods

### 2.1 An In Vivo Guinea Pig Melasma Model and AOPT‐LTL Treatment

A total of 20 female tri‐color guinea pigs (6–8 weeks, 250 ± 15 g) were housed under pathogen‐free conditions, equipped with soft bedding in a standardized environment (temperature 22°C ± 2°C, 12‐h light/dark cycle) and habituated for 1 week. Before the treatment, the back hair of guinea pigs was shaved every 3 days to expose a skin area sizing 5 cm × 5 cm. Guinea pigs were randomly divided into control group and model group, with 10 animals per group, and the back skin of each group was divided into treatment group and nontreatment group: control group, control + AOPT‐LTL group, melasma group, and melasma + AOPT‐LTL group. Briefly, guinea pigs in the melasma and melasma + AOPT‐LTL groups were injected with 7.5 mg/kg progesterone (Jilin Huamu Animal Health Products Co. Ltd) into the hind leg every morning (alternative between the two legs to avoid repeated injections). Since extensive research has confirmed that ultraviolet B (UVB) irradiation alone can successfully establish melasma animal models, and UVB directly activates melanocytes while stimulating keratinocytes to release pro‐melanogenic factors, thereby promoting melanin synthesis and inducing cutaneous hyperpigmentation, we employed UVB for modeling. Additionally, the guinea pigs were exposed to 500 mJ/cm^2^ UVB radiation for 1 h every afternoon in a self‐made irradiation box, with eyes covered and kept 50 cm away from the UVB lamps (JCB35‐24‐01, Sigma, Shanghai, China). The intensity of UVB radiation was measured using an ultraviolet radiometer (Sigma, Shanghai, China), and changes in the back skin were daily observed for 30 days.

AOPT‐LTL treatment was initiated after the modeling of melasma. After the topical application of a transparent and colorless gel on the back skin of the guinea pigs, three sessions of IPL treatment at weekly intervals were performed using a M22 IPL platform (Lumenis Medical Company, USA) (590 nm filter, energy density 10 J/cm^2^, three pulses, pulse width 8‐6‐6 ms, and pulse delay 40 ms). To avoid overstimulating the animal's skin, ensure model stability, and use standardized parameters for mechanism validation, the fluence was fixed at 10 J/cm^2^. Images of the back skin were captured using a digital camera (EOS 90D, Canon, Japan). Skin samples were finally collected one week after the end of the experiment.

### 2.2 Melasma Subjects and AOPT‐LTL Treatments

Twenty patients diagnosed with melasma independently by two dermatologists were included. Those with facial inflammatory disorders (e.g., rosacea, post‐inflammatory hyperpigmentation), scar constitution, other hyperpigmentation disorders, photoelectric or drug treatment within 3 months, and pregnant or lactating women were excluded.

Three sessions of IPL treatment were performed in all patients using an M22 Laser Skin Resurfacing Machine (Lumenis, Israel) with a 590/640 nm filter (energy density range 10–16 J/cm^2^, three pulses, pulse width 8‐6‐6 ms, and pulse delay 35–40 ms). Unlike animal experiments, human treatments must take individual tolerance into account. The fluence range (10–16 J/cm^2^) was dynamically adjusted based on skin phototype (e.g., Fitzpatrick classification), pigment depth, and vascular response, ensuring the treatment endpoint remained “skin temperature ≤ 38.5°C” (to prevent thermal damage) while activating PBM. Before and 1 month after each session, facial images were taken and analyzed using a multispectral facial image processing workstation (Jiangsu Beining Intelligent Technology Development Co. Ltd., China). The severity of melasma was independently evaluated by two senior dermatologists using scoring systems of the Modified Melasma Area and Severity Index (mMASI) and Erythema Index (EI).

### 2.3 Pathological Staining

Skin samples of guinea pigs were fixed in 4% paraformaldehyde for more than 24 h, embedded in paraffin, and cut into sections with a thickness of 3–5 μm. The sections were stained with hematoxylin and eosin (H&E), Masson's trichrome, Fontana‐Masson, and toluidine blue. Finally, six randomly selected fields per sample were observed. In detail, melanin distribution in the skin was visualized by H&E and Fontana‐Masson staining. Collagen fibers distributed in the dermis were assessed by Masson's trichrome, and mast cell infiltration was evaluated through toluidine blue staining. Image‐Pro Plus software (Media Cybernetics Inc.) was used for quantification of pathological changes.

### 2.4 Immunohistochemical and Immunofluorescence Staining

Tissue sections were processed for deparaffinization, antigen retrieval in sodium citrate buffer (pH 6.0) or EDTA buffer (pH 9.0), blockage of endogenous peroxidase activity in 3% hydrogen peroxide solution, and incubation with staining reagents (all Servicebio, Wuhan, China) in 3% bovine serum albumin (BSA). Immunohistochemically stained sections were incubated with the rabbit anti‐CD31 antibody (1:100, Venus Gene, China) at 4°C overnight, immersed in PBST for 5 min × 3 times, and incubated with the goat anti‐rabbit secondary antibody HRP (1:300, Servicebio, China) for 1 h at room temperature. After washing again, sections were stained with DAB, counterstained with hematoxylin, dehydrated, and sealed for observation. Immunofluorescence staining was performed by section incubation with the rabbit anti‐c‐KIT (1:500, Abcam, ab283653, UK) and rabbit anti‐SCF (1:500, Abcam, ab64677, UK) antibodies at 4°C overnight, and immersed in PBST for 5 min × 3 times. The sections were incubated with Cy3‐labeled goat anti‐rabbit secondary antibody and FITC‐labeled goat anti‐rabbit secondary antibody (1:300, Servicebio, China) for 1 h at room temperature. After washing, sections were incubated with DAPI in the dark for 10 min, sealed, and observed under a fluorescence microscope. The fluorescence intensity was statistically analyzed by Image‐Pro Plus software.

### 2.5 Quantitative Reverse Transcription Polymerase Chain Reaction (qRT‐PCR)

Skin samples were lysed in 1 mL of TRIzol (Takara, Shiga, Japan). Total RNA was extracted using a high‐speed cryogenic tissue grinding machine (Servicebio, China) and reversely transcribed into cDNA with a quantity of 2000 ng of RNA using the PrimeScript RT kit (Takara, Shiga, Japan). qRT‐PCR was performed using TB Green Premix Ex Taq II (Takara, Shiga, Japan) on a BIO‐RAD CFX system (BioRad, Munich, Germany). Five replicates were performed for each sample. Relative expression was calculated using the algorithm 2^−ΔΔCt^, with ACTB as an internal reference. The primers were designed by Shanghai Sangon Bioengineering Co. LTD, and their sequences were shown in the Supporting Information.

### 2.6 Western Blotting

Skin samples were lysed in the mixture of RIPA and PMSF (Beyotime Biotechnology, China) at a ratio of 100:1. The total protein was collected and quantified using the BCA Protein Assay Kit (Beyotime Biotechnology, China). Subsequently, proteins were separated by SDS‐PAGE gel electrophoresis, transferred onto PVDF membranes, and immersed in TBST buffer containing 0.05% Tween 20 and 5% BSA. Primary antibodies against c‐KIT (1:5000, Abcam, ab283653, UK), SCF (1:1000, Abcam, ab64677, UK) and β‐actin (1:1000, Abcam, UK) were incubated overnight at 4°C, followed by TBST washing for 10 min × 3 times. Membranes were further incubated with the goat anti‐rabbit HRP (1:1000, Beyotime Biotechnology, China) or the goat anti‐mouse HRP (1:1000, Beyotime Biotechnology, China) secondary antibody for 1 h at room temperature. Finally, protein bands were visualized using a gel imager (Tanon 5200 Multi, Shanghai, China) and quantified with the normalization of β‐actin by Image‐Pro Plus software.

### 2.7 Statistical Analysis

GraphPad Prism 8 (GraphPad Software Inc., San Diego, CA, USA) was used for statistical analysis. Differences were compared by the *t*‐test or one‐way analysis of variance (ANOVA). All normally distributed data were presented as mean ± standard deviation (SD). *p* < 0.05 was considered statistically significant.

## 3 Results

### 3.1 AOPT‐LTL Treatment Reduced Skin Pigmentation in Guinea Pigs With Melasma

**FIGURE 1:**
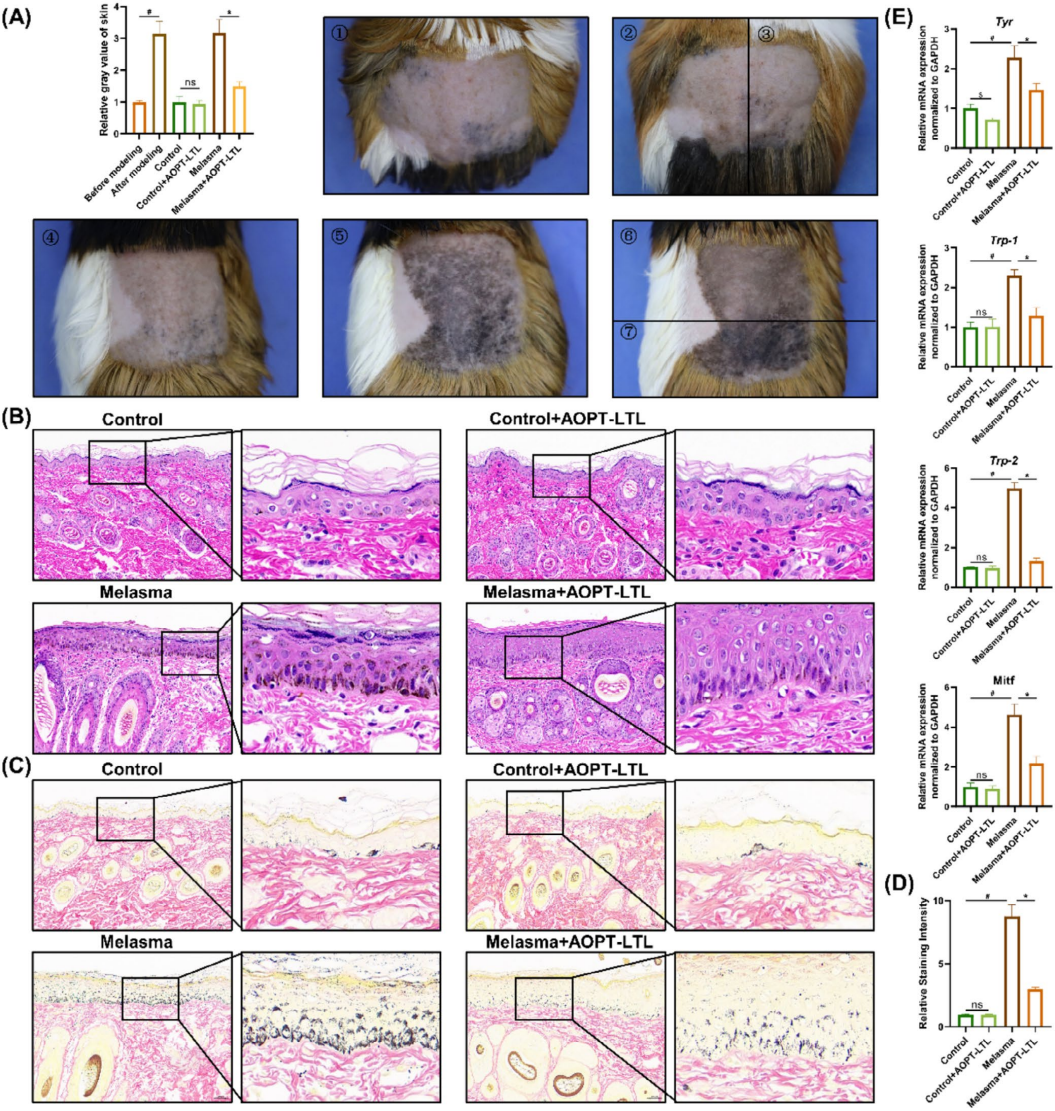
AOPT‐LTL treatment reduces skin pigmentation in guinea pigs with melasma. (A) Gross images of skin pigmentation in guinea pigs and quantification of gray values (1. control group before the modeling; 2. control group; 3. control + AOPT‐LTL group; 4. melasma group before the modeling; 5. melasma group at the end of modeling; 6. melasma group; 7. melasma + AOPT‐LTL group). (B–D) H&E (B) and Fontana‐Masson staining (C, D) of skin tissue (magnification = 20×, scale bar = 200 μm). (E) The mRNA levels of TYR, TRP‐1, TRP‐2 and MITF in skin tissue. *n* = 5, ^#^ *p* < 0.05 vs. control group, *p < 0.05 vs. control + AOPT‐LTL group.

The skin on the UVB‐exposed side of guinea pigs showed deeper color, stronger melanin pigmentation, and gray values did not decrease after 1 month of the modeling, confirming the stability of the melasma model in guinea pigs. After three sessions of AOPT‐LTL treatments, there were significant decreases in skin pigmentation and gray value (*p* < 0.05) (Figure 1A). In comparison to the control group, the melasma group presented evident epidermal hyperplasia and massive deposition of melanin particles in the basal layer of the skin (Figure 1B–D). Notably, the deposition of melanin particles was significantly reduced in the melasma + AOPT‐LTL group (*p* < 0.05). Furthermore, qRT‐PCR revealed significantly higher mRNA levels of tyrosine (TYR), tyrosinase‐related protein 1 (TRP‐1), tyrosinase‐related protein 2 (TRP‐2), and microphthalmia‐associated transcription factor (MITF) in skin tissue of the melasma group than in the control group (*p* < 0.05) (Figure 1E), which were then significantly downregulated by AOPT‐LTL treatment.

### 3.2 AOPT‐LTL Treatment Reduced Mast Cell Infiltration in Guinea Pigs With Melasma

**FIGURE 2:**
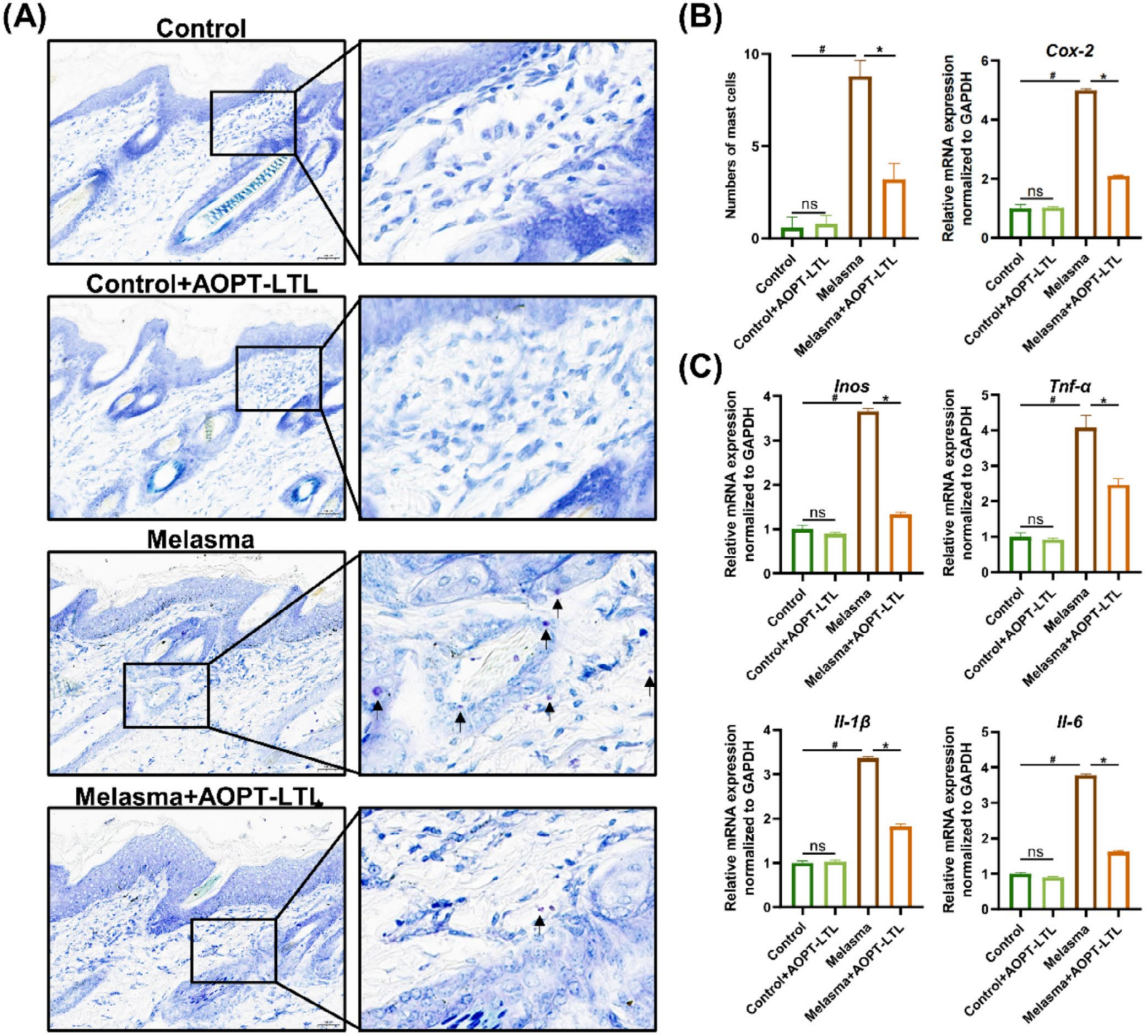
AOPT‐LTL treatment reduces mast cell infiltration in guinea pigs with melasma. (A, B) Toluidine blue staining of mast cell infiltration in skin tissue (A) and the quantification (B) (magnification = 20×, scale bar = 200 μm). (C) The mRNA levels of COX‐2, INOS, TNF‐α, IL‐1β, and IL‐6 in skin tissue. *n* = 5, ^#^ *p* < 0.05 vs. control group, *p < 0.05 vs. control + AOPT‐LTL group.

Infiltration of mast cells was occasionally observed in the control group, but significantly stronger in the melasma group. In contrast, the number of mast cells in the melasma + AOPT‐LTL group was significantly lower than that in the melasma group (*p* < 0.05) (Figure 2A,B). Furthermore, the mRNA levels of inflammatory cytokines like tumor necrosis factor‐α (TNF‐α), interleukin‐1β (IL‐1β), and interleukin‐6 (IL‐6), as well as key enzymes regulating inflammatory mediators such as inducible nitric oxide synthase (iNOS) and cyclooxygenase‐2 (COX‐2), were significantly lower in the melasma + AOPT‐LTL group than in the melasma group (*p* < 0.05) (Figure 2C). Except for COX‐2, the mRNA levels of remaining inflammatory cytokines were comparable between the control group and AOPT‐LTL group (*p* > 0.05).

### 3.3 AOPT‐LTL Treatment Reduced Skin Angiogenesis in Guinea Pigs With Melasma

**FIGURE 3:**
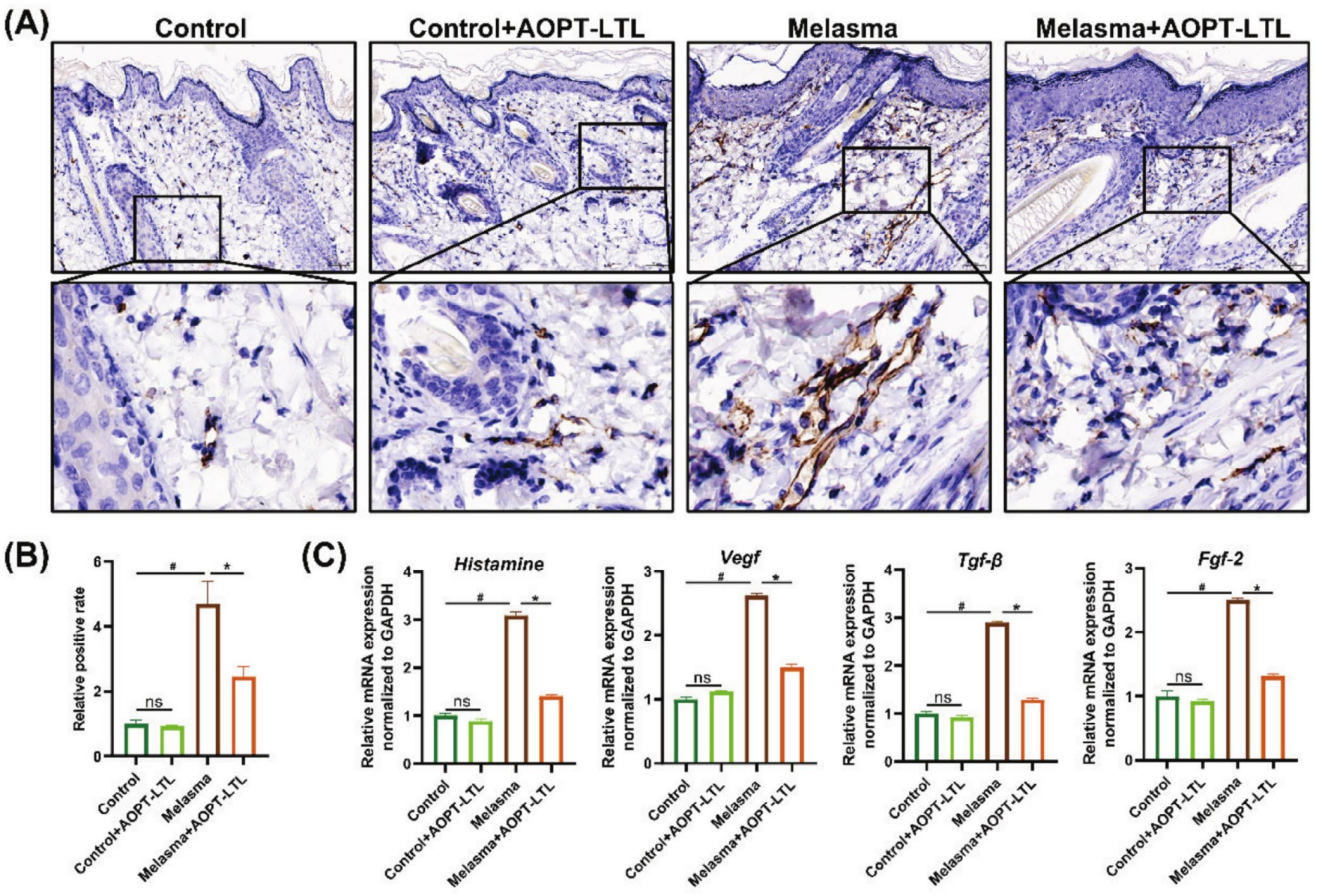
AOPT‐LTL treatment reduces angiogenesis in guinea pigs with melasma. (A, B) Immunohistochemical staining of CD31 in skin tissue (A) and the quantification (B) (magnification = 20×, scale bar = 200 μm). (C) The mRNA levels of VEGF, TGF‐β and FGF‐2 in skin tissue. *n* = 5, ^#^ *p* < 0.05 vs. control group, *p < 0.05 vs. control + AOPT‐LTL group.

CD31 is an established marker for angiogenesis. Immunohistochemical staining visualized notable skin vasodilation and a significantly higher positive rate of CD31 in the melasma group than in the control group (Figure 3A,B). AOPT‐LTL treatment significantly inhibited the amount of blood vessels. Histamine is a vasodilator active mediator released by mast cells. VEGF, TGF‐β and FGF‐2 are cytokines responsible for promoting the proliferation of vascular endothelial cells. Their mRNA levels were significantly higher in the melasma group than in the control group, but were downregulated by AOPT‐LTL treatment (*p* < 0.05) (Figure 3C).

### 3.4 AOPT‐LTL Treatment Relieved Skin Photoaging in Guinea Pigs With Melasma

**FIGURE 4:**
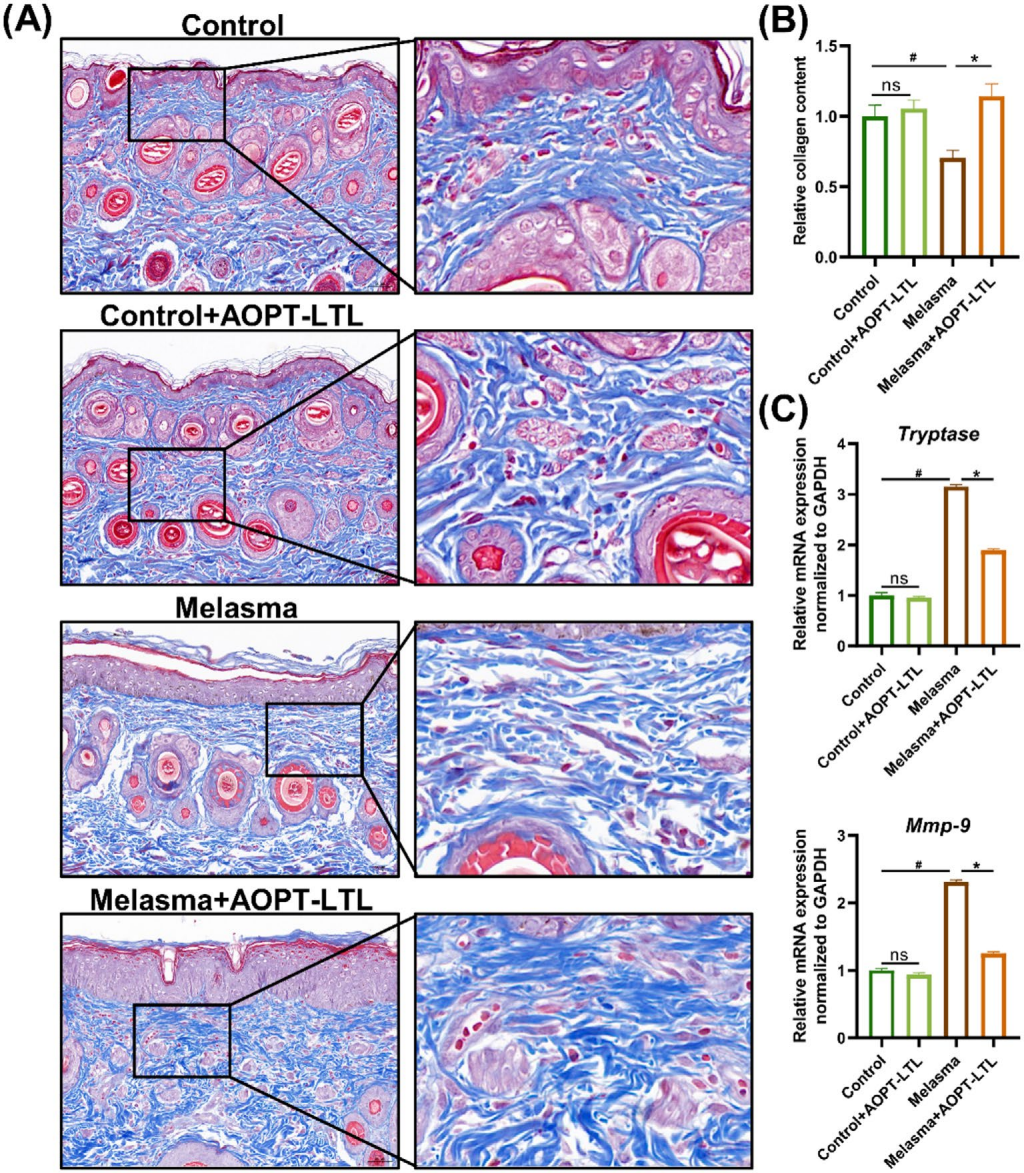
AOPT‐LTL treatment relieves skin photoaging in guinea pigs with melasma. (A, B) Masson's trichrome staining of collagen fibers in skin tissue and the quantification (magnification = 20×, scale bar = 200 μm). (C) The mRNA levels of tryptase and MMP‐9 in skin tissue. *n* = 5, ^#^ *p* < 0.05 vs. control group, *p < 0.05 vs. control + AOPT‐LTL group.

In comparison to the control group, the collagen fiber content fell in the melasma groups, accompanied by an evident disorderly arrangement (*p* < 0.05) (Figure 4A,B). Following the AOPT‐LTL treatment, the number of collagen fibers increased in the dermis, with a more orderly and compact arrangement (*p* < 0.05). Tryptase and matrix metalloproteinase‐9 (MMP‐9) are two enzymes responsible for collagen degradation, and their upregulated mRNA levels in the melasma group were consistently reduced by AOPT‐LTL treatment (Figure 4C).

### 3.5 AOPT‐LTL Treatment May Have Reduced Mast Cell Infiltration in Guinea Pigs With Melasma by Inhibiting the SCF/c‐KIT Ligand/Receptor Pathway

**FIGURE 5:**
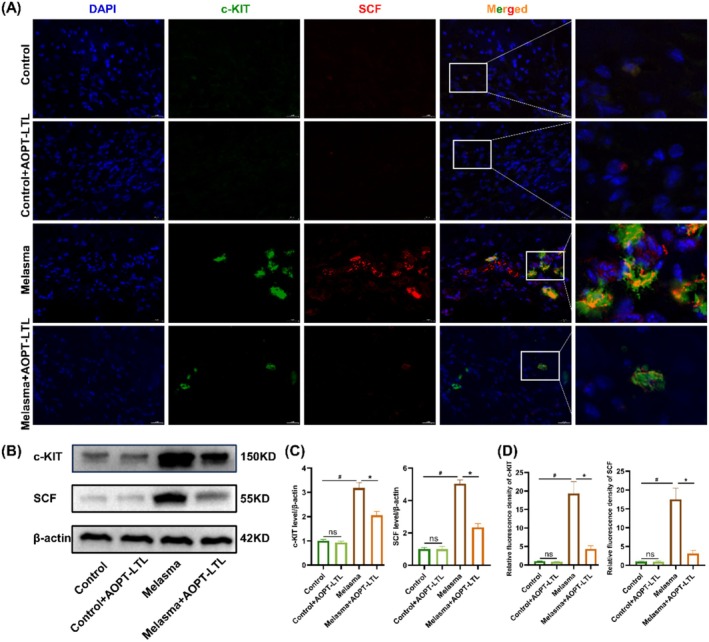
AOPT‐LTL treatment reduces mast cell infiltration in guinea pigs with melasma by inhibiting the SCF/c‐KIT ligand/receptor pathway. (A) Immunofluorescence staining of c‐KIT and SCF in skin tissue (magnification = 20×, scale bar = 200 μm). (B) Protein levels of c‐KIT and SCF in skin tissue. (C, D) The mRNA levels of c‐KIT (C) and SCF (D) in skin tissue. *n* = 5, ^#^ *p* < 0.05 vs. control group, *p < 0.05 vs. control + AOPT‐LTL group.

Compared to the control group, the melasma group showed extensive distribution of green fluorescence‐labeled c‐KIT and the red fluorescence‐labeled SCFs in the dermis. Moreover, c‐KIT and SCF were co‐localized in the cytoplasm. Following AOPT‐LTL treatment, positive expression of c‐KIT and SCF were significantly reduced (*p* < 0.05) (Figure 5A,D). Western blotting consistently showed upregulation of c‐KIT and SCF in dermal cells in the melasma group compared to the control group. Their protein levels were significantly downregulated after AOPT‐LTL treatment (*p* < 0.05) (Figure 5B,C).

### 3.6 AOPT‐LTL Treatment Relieved the Clinical Manifestations of Melasma Patients

**FIGURE 6:**
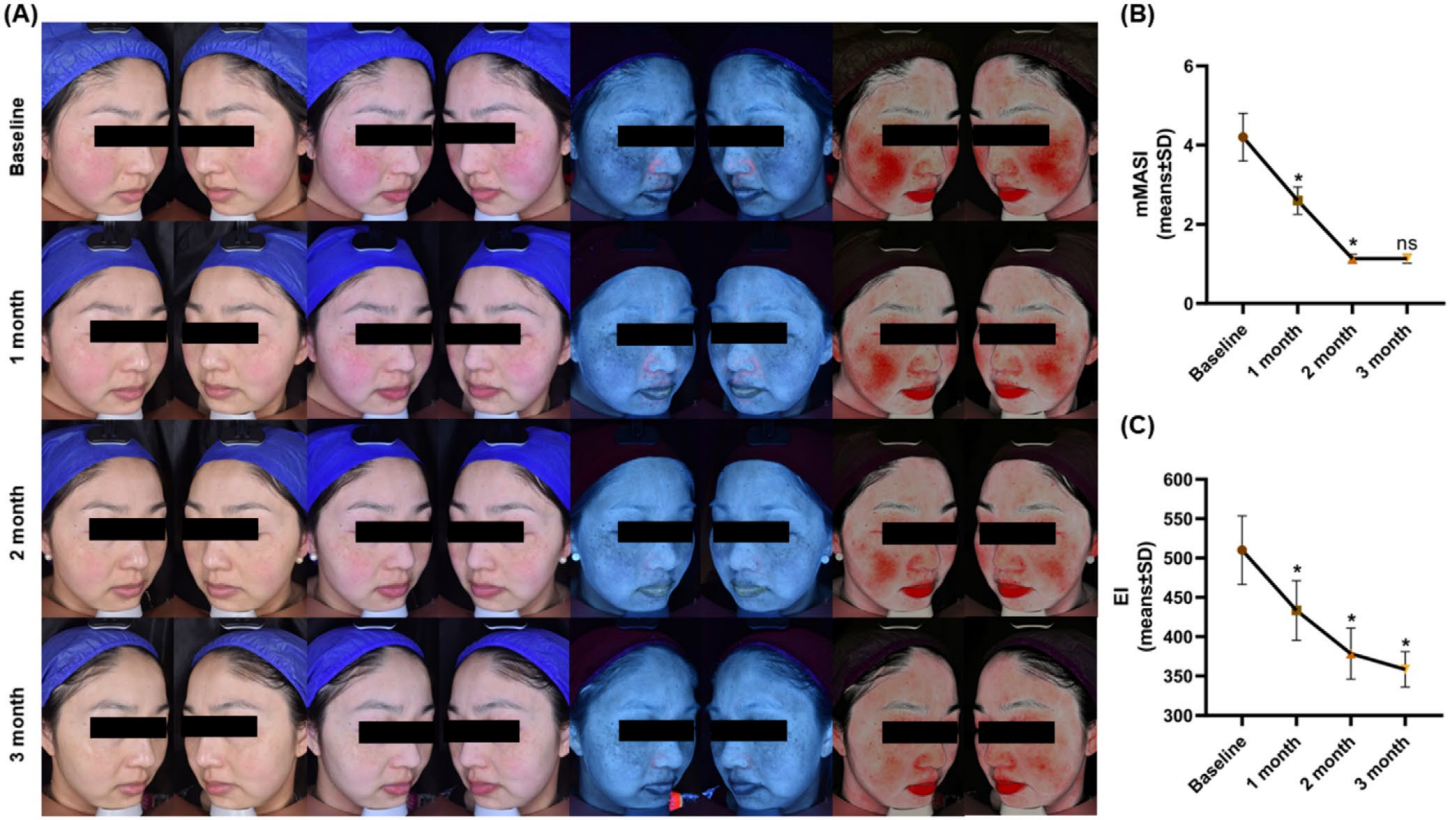
AOPT‐LTL relieves the clinical manifestations of melasma patients. (A) Representative polarized light images (left), ultraviolet pigment images (middle), and red zone images (right) of melasma patients before (baseline) and 1 month after each session of IPL treatment. (B, C) The mMASI (B) and CEA scores (C) of melasma patients before and 1 month after each session of IPL treatment. *n* = 20. *p < 0.05 vs. baseline; ns, no significant difference.

Representative images of a patient with melasma before (baseline) and 1 month after each session were captured (Figure 6A). AOPT‐LTL treatment significantly reduced skin melanin content and erythema severity. In comparison to the 3.20 points at baseline, the mean mMASI score decreased to 1.13 points at 1 month after three sessions of IPL treatment significantly (Figure 6B), and the EI score significantly decreased from 510 points to 358 points (Figure 6C) (both *p* < 0.05). None of the melasma patients experienced significant adverse effects, validating the high safety of the AOPT‐LTL treatment.

## 4 Discussion

**FIGURE 7:**
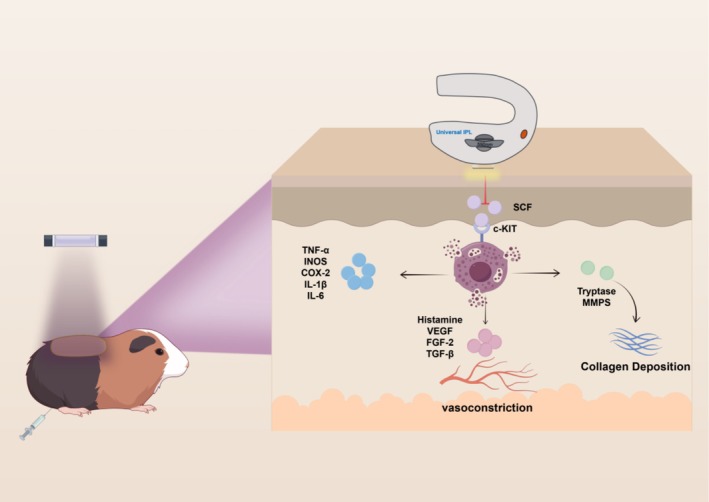
Graphic summary. AOPT‐LTL treatment counters melasma by reducing melanogenesis, inflammation, angiogenesis, mast cell infiltration, and collagen degeneration via inhibiting the SCF/c‐KIT ligand/receptor pathway in the guinea pig model.

Our findings proved that AOPT‐LTL treatment significantly reduced skin pigmentation and erythema severity in both the in vivo guinea pig model and the cohort of melasma patients. The innovative mode of AOPT‐LTL effectively achieved the goal of reducing mast cell infiltration, angiogenesis, and inflammation in the skin tissue of melasma pigs, which may have been through the inhibition of the SCF/c‐KIT ligand/receptor pathway (Figure 7). Overall, three sessions of AOPT‐LTL treatment greatly relieved melasma without significant adverse events.

The low‐energy and large‐spot Q‐switched Nd:YAG laser has been extensively utilized in the treatment of melasma in Asia. It effectively reduces skin pigmentation by targeting melanosomes through the subcellular selective photothermal effect [20]. However, recurrence is common in melasma, as evidenced by a high recurrence rate of 58.8% at one year after treatment [21]. Frequent treatments of the Q‐switched Nd:YAG laser also induce adverse events like mottled hypopigmentosis, due to short pulse width and peak power [22, 23]. Superior to the conventional laser treatment, IPL offers greater control over parameters such as pulse number, pulse width, and pulse delay, allowing more moderate energy output and less irritation to melanocytes [19]. Li et al. [24] reported that IPL (560/590 nm filter, 13–17 J/cm^2^ fluence, dual‐pulse) significantly reduced the mMASI in melasma patients. A retrospective analysis revealed that IPL, using conventional parameters provided by the manufacturer, results in melasma‐like hyperpigmentation in treating pigmented diseases [17]. Although the traditional mode of high energy, short pulse width, and short pulse can achieve the goal of selective thermal decomposition of melanosomes, the high‐intensity heating often causes non‐specific thermal damage [14]. Inflammation accompanied by high‐energy irritation worsens skin pigmentation in melasma patients [25]. Bae et al. [15] discovered that low‐energy IPL (10 or 13 J/cm^2^, dual‐pulse) yields more benefits and fewer side effects in Asian melasma skin. The AOPT‐LTL mode is characterized as low energy, long pulse width, and three pulses. Although there is a lack of direct comparative experiments between AOPT‐LTL and traditional IPL, our preliminary studies indicated that three sessions of AOPT‐LTL treatment effectively prevented thermal damage to surrounding tissues and reduced melanin content and erythema severity.

Mast cell infiltration is a pathological landmark in melasma lesions [8]. Ultraviolet light exposure and psychological stress are the dominant factors driving the recruitment and degranulation of mast cells [26]. Activated mast cells further stimulate melanin synthesis by releasing proteases, eicosanoid acids, and cytokines. Additionally, mast cells contribute to the upregulation of melanin by enhancing the maturation and transport of melanosomes [27, 28, 29]. Through reducing the number of mast cells, tranexamic acid effectively suppresses melasma [30]. Recent evidence shows that IPL treatment alleviates inflammatory responses by inhibiting mast cell degranulation [31]. We consistently observed that AOPT‐LTL treatment significantly reduced mast cell infiltration and the release of pro‐inflammatory cytokines (TNF‐α, IL‐1β, IL‐6) and the expression of key enzymes regulating inflammatory mediators (iNOS, COX‐2) in a guinea pig model of melasma. Overexpression of SCF and its receptor c‐KIT plays a crucial role in the facial pigmentation of melasma [32]. Binding to the c‐KIT receptor on the surface, SCF causes the degranulation of mast cells and the release of active mediators and cytokines associated with melasma [33]. Dai et al. [18] discovered that LED irradiation at 590 nm significantly reduced melanin production by downregulating SCF. In the present study, the AOPT‐LTL treatment significantly inhibited the SCF/c‐KIT ligand/receptor pathway in the skin tissue of guinea pigs with melasma.

Both the number and volume of blood vessels increase within the lesion of melasma. A positive correlation lies between skin pigmentation and the number of blood vessels [7, 34]. The proliferation of blood vessels leads to an influx of inflammatory cells and cytokines (e.g., VEGF, TGF‐β, and FGF‐2) into the skin lesions, further forming a positive feedback loop to promote vascular proliferation in melasma [35]. A case reported in a split‐face controlled study that the vascularization of melasma was remarkably reduced on the side treated with pulsed fuel laser combined with topical medication, without inducing a recurrence within three years, compared with the side treated with topical medication alone, which suggests that targeting blood vessels may play a role in preventing recurrence [36]. LED irradiation inhibits migration and tube formation in human microvascular endothelial cells, thus repressing erythema associated with melasma [18]. It is worth noting that IPL requires a high energy output for targeted coagulation of blood vessels. Studies have demonstrated that blood coagulation can only occur when the temperature exceeds 70°, and the blood vessel wall must be sufficiently damaged [37]. Clearly, our treatment parameters cannot reach the temperature required for blood vessel coagulation. Jiang et al. [31] discovered that rosacea patients benefit from the PBM effect of IPL, with a CEA score significantly reduced after treatment. In this study, we observed that the AOPT‐LTL treatment also effectively inhibited vascular expression in the melasma guinea pig model and significantly decreased EI scores in clinical patients. This may have been attributed to the ability of AOPT‐LTL treatment to effectively suppress vascular endothelial cell proliferation and pro‐angiogenic cytokine expression (VEGF, TGF‐β, FGF‐2). Additionally, it inhibited mast cell numbers and degranulation while reducing histamine release, indirectly promoting blood vessel contraction.

Melasma can be considered a photoaging disorder with evident collagen degradation and loss in the superficial dermis [38]. Genetic profiling reveals upregulation of genes associated with collagen degradation and downregulation of genes associated with collagen synthesis [39]. Xiao et al. [40] proved the efficacy of PF‐GL‐TE gel in reversing skin aging by mitigating collagen loss in rats with melasma. Our findings showed that the AOPT‐LTL treatment significantly increased the number of collagen fibers in the dermis and restored their arrangement by downregulating tryptase and MMP‐9, thereby curbing photoaging in guinea pigs with melasma.

The epidermal thickening we observed in the melasma animal model is contrary to the epidermal thinning found in previous human studies on melasma [41]. This discrepancy deserves in‐depth investigation to clarify the underlying reasons. The animal model in this study was established by progesterone injection combined with UVB irradiation [42, 43]. It is known that chronic UVB exposure induces epidermal hyperplasia, which is a protective response that enhances the skin's physical barrier against further ultraviolet damage by stimulating keratinocyte proliferation [44]. In this animal model, this radiation‐induced acute proliferative effect may be dominant, leading to epidermal thickening. In contrast, the occurrence of human melasma is a persistent pathological state caused by long‐term, cumulative exposure to multiple factors (such as chronic ultraviolet radiation, hormonal fluctuations, genetic susceptibility, and impaired barrier function), which ultimately leads to gradual epidermal atrophy [45]. This difference indicates that although the animal model can effectively simulate pigmentation and inflammatory responses, it may not fully replicate the long‐term structural degradation observed in human melasma. Future studies should verify whether long‐term follow‐up of the animal model will reveal a transition to epidermal thinning, thereby more closely approximating the human pathological state.

Several limitations existed in this experiment. Firstly, although this study has demonstrated that AOPT‐LTL treatment may have improved melanin and erythema levels by reducing mast cell infiltration in vivo, further in vitro experiments are needed to verify the specific mechanism of mast cell inhibition and the causal relationships. Moreover, the absence of a direct head‐to‐head comparison between AOPT‐LTL and conventional IPL warrants dedicated comparative experiments in future studies. Additionally, a larger sample size cohort study of melasma patients with long‐term follow‐up is essential to examine the clinical efficacy of the AOPT‐LTL treatment and the recurrence rate of melasma.

## 5 Conclusion

In summary, three sessions of AOPT‐LTL treatment are effective to treat melasma by reducing melanogenesis, mast cell infiltration, inflammation, angiogenesis, and collagen degeneration. Additionally, it suppresses mast cell proliferation and migration, potentially by modulating the SCF/c‐KIT ligand/receptor pathway.

## Author Contributions

Conceived and designed the experiments: Jing Wang, Xinlan Wang, Li Gu, Bingrong Zhou, Hui Hua. Performed experiments and analyzed data: Jing Wang, Xinlan Wang, Li Gu. Contributed reagents/materials/analysis tool: Zhinan Shi, Zhiyi Xu, Siqi Shen. Investigation: Liqun Gu, Chenglong Jin. Methodology: Lin Chen, Linling Ju. Review and editing: Bingrong Zhou, Hui Hua. Writing: Jing Wang, Xinlan Wang, Li Gu. All authors have approved the submission of this manuscript.

## Ethics Statement

The study was conducted in accordance with the Declaration of Helsinki and was approved by the Ethics Committee of Nantong Third People's Hospital (Ethics number: EL2024024). Informed ConsentInformed consent was obtained from all individual participants included in the study. Patients diagnosed with melasma were recruited in the Nantong Third People's Hospital. Animal studies were approved by the Animal Ethics Committee of Nantong University (Ethics number: S20240215‐031) and followed its guiding principles. Guinea pigs were provided by the Laboratory Animal Center of Nantong University.

## Conflicts of Interest

The authors declare no conflicts of interest.

## Supporting information


**Data S1:** jocd70443‐sup‐0001‐DataS1.xlsx.

## Data Availability

The data that supports the findings of this study are available in the Supporting Information of this article.
